# Young Adult Exposure to Cardiovascular Risk Factors and Risk of Events Later in Life: The Framingham Offspring Study

**DOI:** 10.1371/journal.pone.0154288

**Published:** 2016-05-03

**Authors:** Mark J. Pletcher, Eric Vittinghoff, Anusorn Thanataveerat, Kirsten Bibbins-Domingo, Andrew E. Moran

**Affiliations:** 1 Departments of Epidemiology and Biostatistics, & Medicine, University of California, San Francisco, San Francisco, California, United States of America; 2 Division of General Medicine, Columbia University Medical Center, New York, New York, United States of America; Shanghai Institute of Hypertension, CHINA

## Abstract

**Background:**

It is unclear whether coronary heart disease (CHD) risk factor exposure during early adulthood contributes to CHD risk later in life. Our objective was to analyze whether extent of early adult exposures to systolic and diastolic blood pressure (SBP, DBP) and low-and high-density lipoprotein cholesterol (LDL, HDL) are independent predictors of CHD events later in life.

**Methods and Findings:**

We used all available measurements of SBP, DBP, LDL, and HDL collected over 40 years in the Framingham Offspring Study to estimate risk factor trajectories, starting at age 20 years, for all participants. Average early adult (age 20–39) exposure to each risk factor was then estimated, and used to predict CHD events (myocardial infarction or CHD death) after age 40, with adjustment for risk factor exposures later in life (age 40+). 4860 participants contributed an average of 6.3 risk factor measurements from in-person examinations and 24.5 years of follow-up after age 40, and 510 had a first CHD event. Early adult exposures to high SBP, DBP, LDL or low HDL were associated with 8- to 30-fold increases in later life CHD event rates, but were also strongly correlated with risk factor levels later in life. After adjustment for later life levels and other risk factors, early adult DBP and LDL remained strongly associated with later life risk. Compared with DBP≤70 mmHg, adjusted hazard ratios (HRs) were 2.1 (95% confidence interval: 0.8–5.7) for DBP = 71–80, 2.6 (0.9–7.2) for DBP = 81–90, and 3.6 (1.2–11) for DBP>90 (p-trend = 0.019). Compared with LDL≤100 mg/dl, adjusted HRs were 1.5 (0.9–2.6) for LDL = 101–130, 2.2 (1.2–4.0) for LDL = 131–160, and 2.4 (1.2–4.7) for LDL>160 (p-trend = 0.009). While current levels of SBP and HDL were also associated with CHD events, we did not detect an independent association with early adult exposure to either of these risk factors.

**Conclusions:**

Using a mixed modeling approach to estimation of young adult exposures with trajectory analysis, we detected independent associations between estimated early adult exposures to non-optimal DBP and LDL and CHD events later in life.

## Introduction

High blood pressure and cholesterol are risk factors for coronary heart disease (CHD)[1, 2], and many randomized controlled trials have demonstrated reduced CHD event rates when these risk factors are treated with medications in middle-aged and older adults[3–5]. It remains unclear, however, whether cardiovascular risk factor exposure during young adulthood is important for future CHD risk, and whether treatment at this age might modify risk later in life. While blood pressure guidelines recommend medication to treat high blood pressure throughout life including young adulthood[6, 7], cholesterol guidelines depend primarily on predicted 10-year cardiovascular disease risk to target treatment and thus tend not to recommend cholesterol-lowering medication in young adults with high cholesterol unless cholesterol is extremely high[8].

Several lines of evidence suggest that both high blood pressure and high cholesterol cause lasting cardiovascular damage during young adulthood. Early life risk factors are associated with atherosclerosis[9–12] that persists into middle age[13–15]. Cohorts with very long term follow up have shown that blood pressure and cholesterol measured once during young adulthood predict CHD events much later in life[16–21], but these associations could be wholly attributable to later-life risk factor levels, which usually track with early life levels. Prior analyses of the Framingham Heart Study demonstrated persistent effects of remote exposure to cholesterol and blood pressure on atherosclerosis[22] and CHD events[23], but these studies focused on exposure during middle-age and not young adulthood. A recent analysis of the Framingham Offspring Cohort, which included many young adults at its inception, found that duration of exposure to hyperlipidemia at age 35–55 was associated with CHD events[24], but did not adjust for any other young adult risk factor exposures (e.g., blood pressure) that correlate with hyperlipidemia. No prior studies have teased apart the independent effects of risk factor levels during young adulthood from risk factor levels later in life on the occurrence of CHD events.

We used all available measurements of cardiovascular risk factors (blood pressure and cholesterol) measured repeatedly in the Framingham Offspring Study over 40 years to model complete risk factor trajectories starting at age 20 for all study participants, accounting for the discontinuous effects of time-varying medication use. We used those trajectories to estimate average exposure from age 20–39 (young adulthood), and analyzed associations between average risk factor exposure during young adulthood and CHD events occurring later in life.

## Methods

### Approach, study design and participants

Our approach, which relies on mixed effects modeling, is designed to take advantage of all available risk factor measurements to model age-based risk factor trajectories for each study participant. Mixed models can be used to estimate the latent trajectories underlying the observed values for each participant, and, “borrowing strength” across participants, to extrapolate those trajectories a moderate distance beyond the range of the observed data if trajectories are relatively smooth. Using these methods, we imputed risk factor trajectories for each participant to obtain complete estimated risk factor trajectories for each participant from age 20 years forward. We used these trajectories to estimate average risk factor exposure during young adulthood (age 20–39 years) and then assess the associations of these early exposures with cardiovascular events.

We used measurements and follow up from the Framingham Offspring Study, an institutional review board-approved community-based cohort study. The study was comprised primarily of children of participants in the Framingham Heart Study Original Cohort and their spouses, began examinations and follow up in 1971[25], and has conducted in-person examinations every 4–8 years with continuous follow up for cardiovascular events. We included participants with at least one measurement each, at baseline or during follow-up, of body mass index, blood pressure, lipids and medication use, and without a prior myocardial infarction or stroke who were followed beyond the age of 40 for cardiovascular events. Our analysis of the de-identified dataset, which we accessed through the National Heart, Lung and Blood Institute’s BioLINCC repository[26], was approved by Columbia University’s Institutional Review Board (IRB-AAAI5961).

### Cardiovascular risk factor measurements

Cardiovascular risk factors were measured at seven in-person examinations using standard protocols[27]. Systolic and diastolic blood pressures (SBP and DBP) were measured by a study physician two times while seated. Fasting total cholesterol, high-density lipoprotein cholesterol (HDL) and triglycerides were measured using standard enzymatic methods and low-density lipoprotein cholesterol (LDL) was calculated according to the Friedewald equation[28]; we used only LDL and HDL for this analysis. Height and weight measurements were used to calculate body mass index (BMI) in kg/m^2^. Diabetes status was defined by fasting hyperglycemia (>126 mg/dl) or self-reported diabetes treatment at each examination[26], and assumed always to be present after meeting this definition once. Smoking status (current, past, or never) and cigarette packs/day smoked were assessed by self-report. Current medication lists (either with specific names or more general categories, depending on the exam) were collected at each examination, and used to categorize participants as either using or not using any blood pressure and any cholesterol medication at each examination.

### Cardiovascular events

Incident cardiovascular events were ascertained by passive reporting between visits, active surveillance of local newspaper obituaries, hospital, and mortuary records, and by survey during examination interviews, and adjudicated according to standard protocols. For this analysis, we defined our primary outcome as the occurrence of a CHD event including myocardial infarction (recognized, unrecognized or silent) or CHD death. We censored observation time after the occurrence of a cerebrovascular event including fatal and non-fatal stroke (embolic, hemorrhagic or other) or transient ischemic attack.

### Statistical analysis

We used a series of mixed models to estimate age-based risk factor trajectories and obtain fitted values for each risk factor from age 20 years until the end of observed follow up time for each participant, including for participants whose first Framingham examination was after age 40. Gender-specific age trajectories were flexibly modeled using restricted cubic splines with knots at the 10^th^, 50^th^, and 90^th^ percentiles of the age distribution. We started by modeling BMI trajectories as a function of age and gender, with random effects for participant (intercept plus both age spline components), and used the model to estimate annual best linear unbiased predictions (BLUPs) of BMI for each participant. Age at diabetes onset and first use of blood pressure and cholesterol medication, which could be right, left, or interval-censored, were estimated based on log-normal survival models, with gender and BMI as covariates. We estimated age at subsequent cessation and/or re-initiation of medication use by assuming these events occurred at the midpoint between sequential visits where use differed. Finally, we modeled SBP, DBP, LDL and HDL (log-transforming SBP, LDL and HDL), which are medication-responsive, using fixed effects for age, gender, diabetes, BMI, and medication use (both current and history of), and random effects for participant intercept, age (both spline components), and current medication use (allowing the effect of medication to vary by participant). Smooth trajectories were obtained from the BLUPs using a standard back-transformation for log-transformed outcomes. Cholesterol medication was used in the models for both LDL and HDL, and blood pressure medication in the model for SBP and DBP. Among smokers, packs/day was linearly interpolated between visits, while age at cessation among current smokers at the final visit was estimated using a pooled logistic model.

We then used these individual trajectories to estimate time-weighted average exposures. Specifically, young adult exposure was calculated as the time-weighted average for ages 20–39. We also estimated the time-weighted average from age 40 on (also using estimated trajectories), as well as the “current” measurement (most recent measurement with the last directly measured value carried forward, not using estimated trajectories), and used these as time-varying covariates to adjust for “later life exposure” to risk factors. The inclusion of these multiple ways of measuring later life exposure in our statistical models should allow relatively complete accounting for their effects, and more fully isolate the putative effects of early life exposure, which is the primary goal of this analysis.

We used survival analysis methods to analyze the association between young adulthood risk factors exposure (age 20–39) and CHD events later in life (after age 40). With the origin for time to event set at age 40 years and censoring for fatal and non-fatal stroke and non-CHD deaths, we estimated unadjusted CHD event rates, and then used a series of Cox proportional hazards models (with age as the time scale) to adjust for covariates. All multivariable models were adjusted for sex, calendar year (via spline), BMI, diabetes, years with diabetes, smoking status (current/past/never), pack-years of tobacco exposure (via spline), current use of blood pressure and lipid medications, and other cardiovascular risk factors (SBP, DBP, LDL and HDL). For example, our initial model for early life exposure to LDL cholesterol was adjusted for current levels of SBP, DBP and HDL as well as average exposures to SBP, DBP and HDL from age 20–39 and from age 40 on, and for time-varying exposure to lipid-lowering medications along with the other variables. We then adjusted for later life exposure to the predictor of interest; in the example above, we additionally adjusted for current LDL levels and average exposure from age 40 on.

To examine the robustness and consistency of our findings, we reran statistical analyses restricted to persons with at least one direct risk factor measurement before the age of 40, tested for interactions by gender, excluded persons using blood pressure or lipid medication, used only SBP without DBP and vice-versa, used continuous versions of risk factor exposure variables using splines to account for non-linearity of associations, and explored different versions of the continuous models guided by analyses of collinearity.

We used SAS version 9.3 or Stata version 13 for all analyses.

## Results

Of 5124 total Framingham Offspring Study participants in the BIOLINCC dataset, we excluded 186 with completely missing information on BMI, blood pressure, lipids or medication use, 36 for pre-existing cardiovascular disease, and an additional 42 who did not reach the age of 40 before the end of their observed follow up. The remaining 4860 participants included in the study sample were approximately half male (48%) and ranged from 20–70 years of age at the time of their first examination; 2969 (61%) contributed direct measurements of cardiovascular risk factors at an in-person Framingham examination before the age of 40. Most participants (97%) contributed more than one direct measurement over time (mean 6.3/person, range 1–7), and the average length of uncensored observation time after age 40 was over 20 years (Table 1).

**Table 1:** Framingham Offspring Study Sample Examinations and Observation Period.

Characteristic	Study sample
	N = 4860
Sex, n (%) male	2331 (48%)
Age at first in-person examination, years	
- Mean (SD)	36.5 (10)
- Range	20–70
- <40, n (%)	2969 (61%)
Number of in-person examinations attended	
- Mean (SD)	6.3 (1.5)
- Range	1–7
Length of event observation period after age 40, years	
- Mean (SD)	24.5 (8.5)
- Range	1–37

All available direct measurements were used to estimate trajectories of SBP, DBP, LDL, and HDL from age 20 through the end of follow up for each participant. The Figure in S1 Appendix illustrates fitted trajectories for 20 individual participants in comparison with measured values. By the end of follow up, 1719 participants (35%) were using blood pressure medications and 699 (14%) were using lipid medications. Time-weighted average measurements of SBP, DBP, LDL and HDL from age 20–39 were strongly correlated with measurements later in life (Table A in S1 Appendix). Early adult exposure to SBP, DBP, LDL and HDL were all very strongly associated with each other and with other cardiovascular risk factors (see Table 2, below, for LDL, and Tables B-D for SBP, DBP and HDL in S1 Appendix).

**Table 2:** Characteristics of participants at the beginning of follow up, stratified by early life exposure to LDL.

Characteristics	Time-weighted average LDL from age 20–39, mg/dl				p-value
	< = 100	101–130	131–160	>160	
	N = 846	N = 2,281	N = 1,421	N = 312	
Sex, n (%) male	231 (27%)	997 (44%)	895 (63%)	208 (67%)	< .001
Year, mean +/- SD					
- At beginning of follow-up	1983 +/- 7	1980 +/- 7	1977 +/- 6	1976 +/- 5	< .001
- At end of follow-up	2005 +/- 5	2004 +/- 7	2001 +/- 9	1999 +/- 10	< .001
Age, mean +/- SD, years					
- At beginning of follow-up	41 +/- 2	42 +/- 4	44 +/- 5	45 +/- 6	< .001
- At end of follow-up	62 +/- 8	66 +/- 10	68 +/- 10	68 +/- 11	< .001
Body mass index, mean +/- SD, kg/m^2^					
- At beginning of follow-up	25 +/- 4	27 +/- 5	28 +/- 5	29 +/- 4	< .001
- At end of follow-up	28 +/- 5	30 +/- 6	30 +/- 5	30 +/- 5	< .001
Diabetes, n (%)					
- At beginning of follow-up	8 (1%)	45 (2%)	36 (3%)	24 (8%)	< .001
- At end of follow-up	38 (4%)	234 (40%)	201 (14%)	82 (26%)	< .001
Diabetes duration, mean +/- SD, years					
- At beginning of follow-up	.04 +/- .45	.09 +/- .79	.16 +/- 1.1	.55 +/- 2.2	< .001
- At end of follow-up	.7 +/- 3.7	1.6 +/- 5.2	2.1 +/- 6.2	4.5 +/- 9.3	< .001
Current smoking, n (%)					
- At beginning of follow-up	321 (38%)	1108 (49%)	839 (59%)	211 (68%)	< .001
- At end of follow-up	73 (9%)	235 (10%)	224 (16%)	61 (20%)	< .001
Pack-years tobacco exposure, mean +/- SD					
- At beginning of follow-up	11 +/- 14	14 +/- 18	19 +/- 21	23 +/- 22	< .001
- At end of follow-up	14 +/- 20	19 +/- 24	25 +/- 28	29 +/- 28	< .001
Blood pressure medication, n (%)					
- At beginning of follow-up	42 (5%)	144 (6%)	115 (8%)	41 (13%)	< .001
- At end of follow-up	179 (21%)	742 (33%)	623 (44%)	175 (56%)	< .001
Lipid medication use, n (%)					
- At beginning of follow-up	0 (0%)	1 (<1%)	13 (1%)	20 (6%)	< .001
- At end of follow-up	10 (1%)	148 (6%)	361 (25%)	180 (58%)	< .001
SBP age 20–39, mean +/- SD, mmHg	114 +/- 8	119 +/- 9	124 +/- 10	127 +/- 10	< .001
SBP age 40+, mean +/- SD, mmHg					
- At beginning of follow-up	115 +/- 10	121 +/- 12	126 +/- 12	128 +/- 12	< .001
- At end of follow-up	122 +/- 11	127 +/- 12	131 +/- 12	132 +/- 12	< .001
SBP current*, mean +/- SD, mmHg					
- At beginning of follow-up	115 +/- 13	120 +/- 16	126 +/- 17	127 +/- 17	< .001
- At end of follow-up	122 +/- 17	129 +/- 108	133 +/- 18	135 +/- 17	< .001
DBP age 20–39, mean +/- SD, mmHg	74 +/- 6	77 +/- 7	81 +/- 7	83 +/- 7	< .001
DBP age 40+, mean +/- SD, mmHg					
- At beginning of follow-up	75 +/- 7	79 +/- 8	83 +/- 8	84 +/- 8	< .001
- At end of follow-up	74 +/- 6	77 +/- 7	79 +/- 6	80 +/- 6	< .001
DBP current*, mean +/- SD, mmHg					
- At beginning of follow-up	75 +/- 9	79 +/- 10	82 +/- 10	83 +/- 11	< .001
- At end of follow-up	74 +/- 9	75 +/- 10	76 +/- 10	76 +/- 11	< .001
LDL age 20–39, mean +/- SD, mg/dl	89 +/- 9	116 +/- 8	142 +/- 8	172 +/- 12	< .001
LDL age 40+, mean +/- SD, mg/dl					
- At beginning of follow-up	92 +/- 12	126 +/- 12	153 +/- 12	186 +/- 19	< .001
- At end of follow-up	99 +/- 15	125 +/- 16	146 +/- 17	168 +/- 25	< .001
LDL current*, mean +/- SD, mg/dl					
- At beginning of follow-up	87 +/- 17	119 +/- 17	153 +/- 20	193 +/- 29	< .001
- At end of follow-up	99 +/- 24	123 +/- 25	138 +/- 33	149 +/- 46	< .001
HDL age 20–39, mean +/- SD, mg/dl	54 +/- 11	49 +/- 10	45 +/- 8	42 +/- 7	< .001
HDL age 40+, mean +/- SD, mg/dl					
- At beginning of follow-up	56 +/- 13	51 +/- 12	46 +/- 10	43 +/- 9	< .001
- At end of follow-up	57 +/- 14	51 +/- 13	46 +/- 11	44 +/- 9	< .001
HDL current*, mean +/- SD, mg/dl					
- At beginning of follow-up	56 +/- 15	52 +/- 15	47 +/- 13	45 +/- 12	< .001
- At end of follow-up	60 +/- 17	53 +/- 16	47 +/- 14	45 +/- 12	

SBP–Systolic blood pressure; DBP–Diastolic blood pressure; LDL–Low-density lipoprotein cholesterol; HDL–High-density lipoprotein cholesterol

*—Current values are the most recent direct measurements, with the last value carried forward.

510 participants suffered a first CHD event during follow-up. Unadjusted event rates were 8-fold to 30-fold higher in persons with adverse levels of risk factor exposure during young adulthood (age 20–39 years) compared with persons with optimal levels of exposure (Table 3). These associations were attenuated after adjustment for other CHD risk factors and later life exposure, but remained highly significant for DBP and LDL (Table 3). Compared with DBP≤70 mmHg, adjusted hazard ratios (HRs) for non-optimal DBP levels were 2.1 (95% confidence interval (CI): 0.8–5.7) for DBP = 71–80, 2.6 (0.9–7.2) for DBP = 81–90, and 3.6 (1.2–11) for DBP>90 (p-trend = 0.019). Compared with LDL≤100 mg/dl, adjusted HRs for non-optimal LDL levels were 1.5 (0.9–2.6) for LDL = 101–130, 2.2 (1.2–4.0) for LDL = 131–160, and 2.4 (1.2–4.7) for LDL>160 (p-trend = 0.009). Early adult exposures to SBP and HDL were not independently associated with future CHD events (Table 3).

**Table 3:** Coronary Heart Disease Events in Framingham Participants with Differing Exposure to Risk Factors During Young Adulthood.

Time-weighted average from age 20–39	N	Coronary Heart Disease Events					
		N (%) with an event	Total person-time observed	Unadjusted event rate, per 1000 person-years	Hazard ratio (95% confidence interval)		
					Unadjusted	Adjusted for other risk factors*	Additionally adjusted for later life exposure*†
SBP, mmHg							
≤120	2,452	82 (3.3%)	59,485	1.4	1 (reference)	1 (reference)	1 (reference)
121–140	2,244	375 (17%)	55,731	6.7	4.4 (3.5–5.6)	1.7 (1.2–2.4)	1.3 (0.9–1.8)
141–160	164	53 (32%)	3,755	14	8.3 (5.9–12)	1.4 (0.8–2.4)	1.0 (0.6–1.8)
>160	0	0	0	---	---	---	---
				Trend p-value	< .001	.22	.98
DBP, mmHg							
≤70	637	5 (0.8%)	13801	0.4	1 (reference)	1 (reference)	1 (reference)
71–80	2400	137 (5.7%)	59857	2.2	5.6 (2.3–14)	2.5 (1.0–6.3)	2.1 (0.8–5.7)
81–90	1588	291 (18%)	40037	7.3	16 (6.7–39)	3.4 (1.3–8.9)	2.6 (0.9–7.2)
>90	235	77 (33%)	5276	1.5	31 (12–77)	5.3 (1.9–14)	3.6 (1.2–11)
				Trend p-value	< .001	< .001	.019
LDL, mg/dl							
≤100	846	19 (2.2%)	19,355	1.0	1 (reference)	1 (reference)	1 (reference)
101–130	2,281	154 (6.8%)	57,319	2.7	2.4 (1.5–3.9)	1.6 (1.0–2.6)	1.5 (0.9–2.6)
131–160	1,421	247 (17%)	34,763	7.1	6.0 (3.8–9.7)	2.7 (1.6–4.4)	2.2 (1.2–4.0)
>160	312	90 (29%)	7,534	12	10 (6.1–16.5)	3.4 (2.0–5.7)	2.4 (1.2–4.7)
				Trend p-value	< .001	< .001	.009
HDL, mg/dl							
>65	328	9 (2.7%)	8,011	1.1	1 (reference)	1 (reference)	1 (reference)
51–65	1,673	74 (4.4%)	42,430	1.7	1.5 (0.7–2.9)	1.0 (0.5–2.0)	1.0 (0.5–2.1)
36–50	2,494	342 (14%)	60,497	5.7	4.7 (2.4–9.1)	1.6 (0.8–3.1)	1.2 (0.5–2.5)
≤35	365	85 (23%)	8,033	11	9.3 (4.7–18)	2.2 (1.1–4.4)	1.4 (0.6–3.2)
				Trend p-value	< .001	.013	.43

SBP–Systolic blood pressure; LDL–Low-density lipoprotein cholesterol; HDL–High-density lipoprotein cholesterol

*—All multivariable models adjusted for age (via Cox model), sex, calendar year (via spline), body mass index, diabetes, years with diabetes, smoking status (current/past/never), pack-years of tobacco exposure (via spline), and use of blood pressure and lipid medications. Models are also adjusted for the *other* risk factors in the table; for example, each adjusted SBP model is adjusted for all the DBP, LDL, HDL predictors including both early and later life exposure.

† - Adjusted for measurements of each risk factor made later in life, including the time-weighted average from age 40+ as well as the most recent measurement (with the last value carried forward), categorized in the same way. For example, the LDL hazard ratios in the last column are adjusted for all other risk factors* as well as LDL exposure from age 40+ as well as the most recent LDL level.

**Fig 1:**
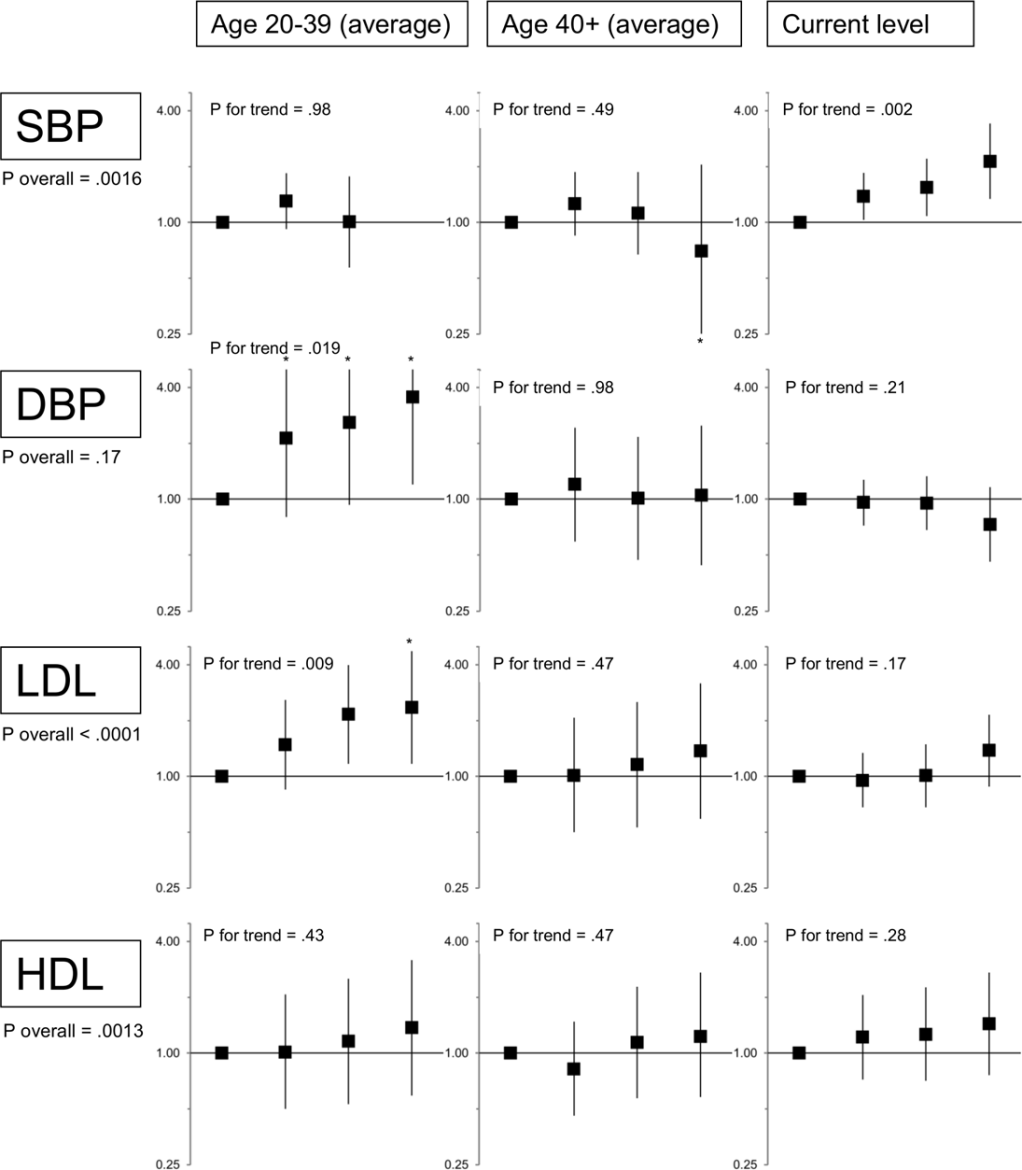
Adjusted associations between SBP, DBP, LDL and HDL levels at different ages and CHD events. Hazard ratios (95% confidence intervals) are adjusted for age (via Cox model), sex, calendar year (via spline), body mass index, diabetes, years with diabetes, smoking status (current/past/never), pack-years of tobacco exposure (via spline), and use of blood pressure and lipid medications. The first column of results (for age 20–39) corresponds to the right-hand column of Table 2. Categories for systolic blood pressure (SBP) are ≤120 (reference), 121–140, 141–160 and >160 mmHg; for diastolic blood pressure (DBP) are ≤80, 81–90, 91–100, and >100; for low-density lipoprotein cholesterol (LDL) are ≤100 (reference), 101–130, 131–160 and >160 mg/dl; and for high-density lipoprotein cholesterol (HDL) are >65 (reference), 51–65, 36–50, and ≤35 mg/dl. “P Overall” refers to a test of the overall contribution of the risk factor (including early, later, and current exposures) to the model. No participants had an average SBP>160 mmHg from age 20–39. The * indicates a truncated confidence interval.

In the fully adjusted model, current SBP was also strongly associated with CHD events; early adult SBP was not (Fig 1). While none of the three HDL variables were independently associated with CHD events in the full model (p-trend = 0.28–0.47), a test of the overall contribution of all HDL variables to the model was highly significant (p = 0.0013, Fig 1). When only the young adult HDL variable was included in the model, it was highly significant (Table 3); the same is true for current HDL (HRs 1.3–2.1, p-trend < .001). In the full model, the two later life LDL variables (time-weighted average after age 40, and current level) were not independently associated with the outcome (Fig 1), but these variables were strongly correlated (Table A in S1 Appendix), and a joint test of significance for both of these later life LDL variables was borderline significant (p = .053). When we dropped the time-weighted average after age 40 variable, the current level variable became significantly associated with the outcome (p for trend = 0.017), and the early life LDL association became even stronger (p for trend = .0005).

Sensitivity analyses demonstrated consistent patterns of association. When we limited our analysis to only the 2969 participants with at least one direct risk factor measurements before age 40, point estimates were similar or even larger for early adult DBP (HRs 1.5–4.4, p-trend = 0.03) and LDL (HRs 2.1–3.0, p-trend = 0.05) and remained null for early adult SBP and HDL (see Table E in S1 Appendix for a full tabulation of results). When early adult DBP was omitted from the model, early adult SBP remained only marginally associated with CHD events (HRs 1.3–1.4, p-trend = 0.39). Among the 2667 participants who never reported using blood pressure or lipid medications, early adult LDL remained significantly associated with CHD events (HRs 1.3–3.4, p-trend = 0.03) but DBP did not, although the HRs were similar in magnitude (HRs 1.2–2.6, p-trend = 0.52); only 12 participants in this subgroup had early adult DBP>90. Among early adult risk factors, only DBP interacted significantly with gender (p < .001); but only 55 men had early adult DBP< = 70, and none of these men had CHD events. When this variable was recoded collapsing categories < = 70 with 71–80, no interaction was noted (p = 0.52). In a model containing continuous instead of categorical risk factors, we found no evidence of non-linearity in any of the predictors, and the significant predictors were the same: current SBP HR = 1.3 (1.1–1.6) per 20 mmHg, early adult DBP HR = 1.6 (1.1–2.3) per 10 mmHg, and early adult LDL HR = 1.5 (1.1–2.0) per 30 mg/dl. Using continuous predictors in the 2969 participants with at least one direct risk factor measurement before age 40 yielded similar results after modifying the analysis to account for collinearity (Table F in S1 Appendix).

## Discussion

In this analysis of the Framingham Offspring Study, we estimated trajectories of SBP, DBP, LDL and HDL during early adulthood and later life, and found early adult exposures to DBP and LDL to be associated with subsequent CHD events. These associations were independent of later life exposures and current risk factor levels. SBP and HDL were also associated with CHD events, but in contrast to DBP and LDL, we could not confirm that exposure to SBP or HDL early in life contributes independently to CHD events.

Our finding that DBP and LDL exposure early in life is associated with CHD events extends a line of inquiry on blood pressure, cholesterol and CHD that began in the 1950’s with early analyses of the Framingham Heart Study’s Original Cohort[29, 30]. Findings in adults led to studies of children and young adults showing associations between these risk factors and atherosclerosis measured by autopsy[9, 12, 31, 32] and non-invasive testing[10, 11, 15, 33–36]. Prior longitudinal analyses of the Framingham Heart Study[22], Atherosclerotic Risk in Community (ARIC) Study[37], and the Coronary Artery Risk Development in Young Adults (CARDIA) Study[13, 14, 38] suggest that atherosclerotic damage may accumulate and persist over many years into middle and late adulthood, and that early life risk factor exposure may be associated with later life atherosclerosis independent from later life exposure levels[13, 14]. Long-term follow-up studies have demonstrated associations between blood pressure and cholesterol measured once during young adulthood with CHD events later in life[16–21], but whether this association is independent of later life risk factor levels has remained unclear. A recent analysis of the Framingham Offspring Cohort, which included many young adults at its inception, found that duration of exposure to hyperlipidemia at age 35–55 was associated with CHD events[24], but did not adjust for any other young adult risk factor exposures (e.g., blood pressure) that strongly correlate with hyperlipidemia (Table 2). Because of strong correlations between risk factors and within risk factor across the life course (“tracking”), teasing apart the effects of early versus later life exposure to each individual risk factor is difficult. Our analysis, which used direct risk factor measurements from over 30,000 study visits and nearly 120,000 person-years of follow up for first CHD events accrued by the Framingham Offspring Study, allowed us to tease apart these factors and detect independent associations between DBP and LDL exposure during early adulthood and CHD events later in life.

Our finding that DBP, not SBP, was a predictor of later life CHD risk is consistent with a previous Framingham analysis showing that DBP is a stronger predictor of events early in life than SBP[39]. Those investigators proposed that different pulse-wave reflection effects on SBP in normotensive and hypertensive young adults obscured the association of SBP with CHD risk in adults <50 years old, a mechanism that changes with stiffening of the arteries later in life. Later in life, both our and prior analyses[39] show SBP to be a strong predictor of events. Because few participants experienced raised SBP during young adulthood, we may also have lacked statistical power to make reliable inference about the association of SBP exposure during ages 20–39 years with later life risk.

Our results are also consistent with recent genetic analyses that bolster the case for a lasting, causal effect of early life exposure. These studies demonstrate that lifelong reductions in blood pressure and cholesterol starting at birth due to genetic variation lead to larger reductions in CHD risk than would be predicted from the degree of differences in risk factor levels later in life[40–43]. These effects are consistent both for single genes (e.g., lower LDL from the R46L allele in PCSK9[40, 41]) and for multivariate genetic risk factor scores for both blood pressure[42] and cholesterol[40, 41, 43]. In contrast, and consistent with our results, a multi-polymorphism score for HDL was not independently associated with CHD[43].

Our analytic methods allow us to estimate risk factor trajectories using all available data starting at age 20 and through the end of follow up, but they are imperfect. Extrapolating backwards to age 20 years is prone to measurement error and may be biased by failure to adequately specify cohort and secular effects (though calendar year was included in all adjusted models). If we assume that our measurement error is mostly non-differential and trajectory estimates for persons with relatively fewer or no measurements before the age of 40 are subject to extra “shrinkage” towards the sample means, our point estimates of effect are likely biased towards the null[44], and perhaps variably so (participants with more direct measurements earlier in life should have less measurement error in the young adult average exposure estimate). However, using all participants despite variable measurement error and borrowing strength across participants using our mixed modeling approach appears to have provided adequate statistical power to detect the presence of an independent association between early adult risk factor exposure and later life CHD risk factors that would have been hard to detect without these methods. We believe our results provide some evidence in support of the *presence* of an early adult exposure effect on later life CHD events, but are likely biased from regression dilution and should not be taken as an accurate estimate of the *size* of such an effect. Additional methodologic work is needed to validate the approach we have taken and account for variable measurement error and regression dilution. There is also the real possibility of residual confounding. We attempted to adjust systematically for potential confounders, but residual confounding from imperfect measurement or unmeasured factors remains a possible explanation for our results. For example, exposure to blood pressure and lipids even *before* age 20 could confound our results if childhood (or even prenatal) exposure to these risk factors is the true driver of later life atherosclerosis[45, 46]; we did not have measurements before age 20 and could not therefore adjust for these factors. Our analysis was focused on detecting associations with early life exposure to risk factors that were independent of later life risk factor exposure, and not on quantifying associations with later life risk factors. By including two versions of later life risk factor exposure in our models, we helped ensure isolation of early life exposure, but simultaneously made it difficult to quantify the independent associations with later life exposure because of collinearity. Thus, the lack of an association between later life LDL and outcomes in our primary outcome model (Fig 1) should not be taken as evidence of an absence of association, especially given very strong prior evidence to the contrary[3, 4] and the results of our sensitivity analysis dropping one of the two later life LDL variables and the joint association test (as described in the Results), which both support the expected association with later life LDL. As with all Framingham-based analyses, the lack of racial and ethnic diversity in the cohort limits our ability to make inferences about non-White populations.

If our findings truly indicate causal and modifiable effects of early life risk factor exposure, they could have important implications. Though current hypertension guidelines recommend treating elevated DBP in young adults, current guidelines for treatment of high cholesterol recommend first estimating medium-term (10-year) atherosclerotic cardiovascular disease risk and recommend statin treatment only when risk is greater than 7.5% in 10 years[8]. These recommendations effectively exclude treatment of young adults unless LDL is extremely high (e.g., >190 mg/dl). If our findings are true, however, young adulthood may be an important window during which LDL-lowering could reduce CHD events later in life. Benefits from treatment, however, would be extremely difficult to confirm. Randomized controlled trials of statin treatment in young adults would require large sample sizes and very long follow-up to detect any delayed effects of statins, and would need to “compete” with later life statin initiation, which is clearly beneficial in many persons[4, 8]. It is unclear, also, whether potential benefits of pharmacotherapy accruing far in the future would be valued by young patients concerned about immediate downsides including cost, side effects, convenience, and other factors that might reduce quality of life. Modeling can help clarify these theoretical tradeoffs, and additional observational studies including diverse participants should be analyzed to confirm our results. Meanwhile, current guidelines strongly recommend optimizing risk factors by maintaining a heart-healthy lifestyle throughout life, including young adulthood[8, 47]; our findings suggest that doing so may indeed provide benefits later in life.

## Supporting Information

S1 Appendix Included in a separate file. (DOC)
