# Supplementary material for: Young Adult Exposure to Cardiovascular Risk Factors and Risk of Events Later in Life: The Framingham Offspring Study
Source: PLoS One. 2016 May 3;11(5):e0154288. doi: 10.1371/journal.pone.0154288 (PMC4854462; doi:10.1371/journal.pone.0154288)

#### Appendix

| **Appendix Table A. Correlations between early life and later life exposure to risk factors in the last year of follow up** | | | |
| --- | --- | --- | --- |
| Exposure variable* | Pearson correlation coefficient (r) | | |
|  | SBP_20-39_ | SBP_40+_ | SBP_current_ |
| SBP_20-39_ | 1 | -- | -- |
| SBP_40+_ | 0.70 | 1 | -- |
| SBP_current_ | 0.41 | 0.83 | 1 |
|  |  |  |  |
|  | DBP_20-39_ | DBP_40+_ | DBP_current_ |
| DBP_20-39_ | 1 | -- | -- |
| DBP_40+_ | 0.71 | 1 | -- |
| DBP_current_ | 0.24 | 0.73 | 1 |
|  |  |  |  |
|  | LDL_20-39_ | LDL_40+_ | LDL_current_ |
| LDL_20-39_ | 1 | -- | -- |
| LDL_40+_ | 0.80 | 1 | -- |
| LDL_current_ | 0.47 | 0.82 | 1 |
|  |  |  |  |
|  | HDL_20-39_ | HDL_40+_ | HDL_current_ |
| HDL_20-39_ | 1 | -- | -- |
| HDL_40+_ | .89 | 1 | -- |
| HDL_current_ | .76 | .93 | 1 |

* - Exposure is estimated for each risk factor in terms of a time-weighted average between age 20-39 years and after the age of 40. Current values are the most recent direct measurements, with the last value carried forward.

| **Appendix Table B. Characteristics of participants at the beginning of follow up, stratified by early life exposure to SBP** | | | | |
| --- | --- | --- | --- | --- |
| Characteristics | Time-weighted average SBP from age 20-39, mg/dl | | | p-value |
|  | <=120  N=2452 | 121-140  N=2244 | 141-160  N=164 |  |
| Sex, n (%) male | 582 (24%) | 1598 (71%) | 151 (92%) | <.001 |
| Year, mean +/- SD |  |  |  |  |
| - At beginning of follow-up | 1982 +/- 7 | 1977 +/- 6 | 1974 +/- 4 | <.001 |
| - At end of follow-up | 2005 +/- 5 | 2001 +/- 9 | 1996 +/- 10 | <.001 |
| Age, mean +/- SD, years |  |  |  |  |
| - At beginning of follow-up | 41 +/- 3 | 44 +/- 5 | 48 +/- 7 | <.001 |
| - At end of follow-up | 65 +/- 9 | 68 +/- 10 | 70 +/- 11 | <.001 |
| Body mass index, mean +/- SD, kg/m^2^ |  |  |  |  |
| - At beginning of follow-up | 25 +/- 4 | 28 +/- 5 | 32 +/- 5 | <.001 |
| - At end of follow-up | 29 +/- 5 | 30 +/- 6 | 32 +/- 6 | <.001 |
| Diabetes, n (%) |  |  |  |  |
| - At beginning of follow-up | 17 (1%) | 81 (4%) | 15 (9%) | <.001 |
| - At end of follow-up | 119 (5% | 391 (17%) | 45 (27% | <.001 |
| Diabetes duration, mean +/- SD, years |  |  |  |  |
| - At beginning of follow-up | .04 +/- .52 | .19 +/- 1.1 | .79 +/- 2.8 | <.001 |
| - At end of follow-up | .70 +/- 3.5 | 2.7 +/- 6.8 | 5.0 +/- 10 | <.001 |
| Current smoking, n (%) |  |  |  |  |
| - At beginning of follow-up | 1085 (44%) | 1278 (57%) | 116 (71%) | <.001 |
| - At end of follow-up | 232 (9%) | 334 (15%) | 27 (16%) | <.001 |
| Pack-years tobacco exposure, mean +/- SD |  |  |  |  |
| - At beginning of follow-up | 12 +/- 15 | 19 +/- 21 | 27 +/- 27 | <.001 |
| - At end of follow-up | 17 +/- 21 | 24 +/- 28 | 33 +/- 32 | <.001 |
| Blood pressure medication, n (%) |  |  |  |  |
| - At beginning of follow-up | 69 (3%) | 229 (10%) | 44 (27%) | <.001 |
| - At end of follow-up | 336 (14%) | 1239 (55%) | 144 (88%) | <.001 |
| Lipid medication use, n (%) |  |  |  |  |
| - At beginning of follow-up | 7 (<1%) | 23 (1%) | 4 (2%) | <.001 |
| - At end of follow-up | 230 (9%) | 430 (19%) | 39 (24%) | <.001 |
| SBP age 20-39, mean +/- SD, mmHg | 112 +/- 5 | 127 +/- 5 | 144 +/- 3 | <.001 |
| SBP age 40+, mean +/- SD, mmHg |  |  |  |  |
| - At beginning of follow-up | 113 +/- 7 | 129 +/- 8 | 150 +/- 9 | <.001 |
| - At end of follow-up | 121 +/- 10 | 133 +/- 10 | 147 +/- 11 | <.001 |
| SBP current*, mean +/- SD, mmHg |  |  |  |  |
| - At beginning of follow-up | 112 +/- 9 | 130 +/- 13 | 159 +/- 20 | <.001 |
| - At end of follow-up | 123 +/- 17 | 135 +/- 17 | 142 +/- 21 | <.001 |
| DBP age 20-39, mean +/- SD, mmHg | 73 +/- 4 | 83 +/- 4 | 92 +/- 4 | <.001 |
| DBP age 40+, mean +/- SD, mmHg |  |  |  |  |
| - At beginning of follow-up | 74 +/- 5 | 85 +/- 6 | 96 +/- 7 | <.001 |
| - At end of follow-up | 74 +/- 5 | 81 +/- 6 | 88 +/- 7 | <.001 |
| DBP current*, mean +/- SD, mmHg |  |  |  |  |
| - At beginning of follow-up | 73 +/- 7 | 84 +/- 9 | 98 +/- 13 | <.001 |
| - At end of follow-up | 74 +/- 8 | 77 +/- 10 | 79 +/- 13 | <.001 |
| LDL age 20-39, mean +/- SD, mg/dl | 114 +/- 22 | 130 +/- 22 | 144 +/- 21 | <.001 |
| LDL age 40+, mean +/- SD, mg/dl |  |  |  |  |
| - At beginning of follow-up | 123 +/- 27 | 137 +/- 27 | 147 +/- 25 | <.001 |
| - At end of follow-up | 126 +/- 26 | 132 +/- 25 | 136 +/- 24 | <.001 |
| LDL current*, mean +/- SD, mg/dl |  |  |  |  |
| - At beginning of follow-up | 120 +/- 32 | 136 +/- 34 | 146 +/- 31 | <.001 |
| - At end of follow-up | 125 +/- 32 | 126 +/- 33 | 120 +/- 32 | .06 |
| HDL age 20-39, mean +/- SD, mg/dl | 52 +/- 10 | 45 +/- 9 | 41 +/- 7 | <.001 |
| HDL age 40+, mean +/- SD, mg/dl |  |  |  |  |
| - At beginning of follow-up | 54 +/- 12 | 46 +/- 11 | 42 +/- 9 | <.001 |
| - At end of follow-up | 55 +/- 13 | 46 +/- 12 | 43 +/- 10 | <.001 |
| HDL current*, mean +/- SD, mg/dl |  |  |  |  |
| - At beginning of follow-up | 54 +/- 14 | 47 +/- 14 | 44 +/- 13 |  |
| - At end of follow-up | 57 +/- 16 | 47 +/- 15 | 43 +/- 14 | <.001 |

* - Current values are the most recent direct measurements, with the last value carried forward.

SBP – Systolic blood pressure; DBP – Diastolic blood pressure; LDL – Low-density lipoprotein cholesterol; HDL – High-density lipoprotein cholesterol

| **Appendix Table C. Characteristics of participants at the beginning of follow up, stratified by early life exposure to DBP** | | | | | |
| --- | --- | --- | --- | --- | --- |
| Characteristics | Time-weighted average DBP from age 20-39, mg/dl | | | | p-value |
|  | <=70  N=637 | 71-80  N=2400 | 81-90  N=1588 | >90  N=235 |  |
| Sex, n (%) male | 55 (9%) | 945 (39%) | 1118 (70%) | 213 (91%) | <.001 |
| Year, mean +/- SD |  |  |  |  |  |
| - At beginning of follow-up | 1985 +/- 7 | 1980 +/- 7 | 1977 +/- 6 | 1975 +/- 4 | <.001 |
| - At end of follow-up | 2006 +/- 4 | 2004 +/- 6 | 2001 +/- 9 | 1996 +/- 10 | <.001 |
| Age, mean +/- SD, years |  |  |  |  |  |
| - At beginning of follow-up | 40 +/- 1 | 42 +/- 4 | 44 +/- 5 | 47 +/- 7 | <.001 |
| - At end of follow-up | 61 +/- 8 | 66 +/- 9 | 68 +/- 11 | 69 +/- 11 | <.001 |
| Body mass index, mean +/- SD, kg/m^2^ |  |  |  |  |  |
| - At beginning of follow-up | 23 +/- 3 | 26 +/- 4 | 29 +/- 5 | 32 +/- 5 | <.001 |
| - At end of follow-up | 27 +/- 4 | 29 +/- 5 | 31 +/- 6 | 31 +/- 6 | <.001 |
| Diabetes, n (%) |  |  |  |  |  |
| - At beginning of follow-up | 4 (0.6%) | 23 (1.0%) | 66 (4%) | 20 (9%) | <.001 |
| - At end of follow-up | 14 (2%) | 166 (7%) | 304 (19%) | 71 (30%) | <.001 |
| Diabetes duration, mean +/- SD, years |  |  |  |  |  |
| - At beginning of follow-up | .04 +/- .6 | .05 +/- .6 | .22 +/- 1.2 | .64 +/- 2.4 | <.001 |
| - At end of follow-up | .3 +/- 2.2 | 1.0 +/- 4.2 | 3.1 +/- 7.4 | 4.7 +/- 8.7 | <.001 |
| Current smoking, n (%) |  |  |  |  |  |
| - At beginning of follow-up | 264 (41%) | 1148 (48%) | 906 (57%) | 161 (69%) | <.001 |
| - At end of follow-up | 70 (11%) | 253 (11%) | 231 (15%) | 39 (17%) | <.001 |
| Pack-years tobacco exposure, mean +/- SD |  |  |  |  |  |
| - At beginning of follow-up | 11 +/- 12 | 14 +/- 16 | 19 +/- 22 | 25 +/- 26 | <.001 |
| - At end of follow-up | 15 +/- 18 | 19 +/- 23 | 25 +/- 28 | 30 +/- 30 | <.001 |
| Blood pressure medication, n (%) |  |  |  |  |  |
| - At beginning of follow-up | 9 (1.4%) | 75 (3.1%) | 190 (12%) | 68 (29%) | .002 |
| - At end of follow-up | 18 (3%) | 494 (21%) | 1005 (63%) | 202 (86%) | <.001 |
| Lipid medication use, n (%) |  |  |  |  |  |
| - At beginning of follow-up | 2 (0.3%) | 11 (0.5%) | 16 (1.0%) | 5 (2.1%) | <.001 |
| - At end of follow-up | 32 (5%) | 278 (12%) | 342 (22%) | 47 (20%) | <.001 |
| SBP age 20-39, mean +/- SD, mmHg | 107 +/- 4 | 117 +/- 5 | 128 +/- 5 | 141 +/- 5 | <.001 |
| SBP age 40+, mean +/- SD, mmHg |  |  |  |  |  |
| - At beginning of follow-up | 106 +/- 6 | 118 +/- 7 | 130 +/- 4 | 145 +/- 10 | <.001 |
| - At end of follow-up | 114 +/- 9 | 125 +/- 10 | 134 +/- 10 | 144 +/- 11 | <.001 |
| SBP current*, mean +/- SD, mmHg |  |  |  |  |  |
| - At beginning of follow-up | 105 +/- 8 | 117 +/- 10 | 131 +/- 14 | 153 +/- 21 | <.001 |
| - At end of follow-up | 114 +/- 15 | 127 +/- 17 | 135 +/- 17 | 142 +/- 20 | <.001 |
| DBP age 20-39, mean +/- SD, mmHg | 67 +/- 2 | 75 +/- 3 | 84 +/- 3 | 93 +/- 2 | <.001 |
| DBP age 40+, mean +/- SD, mmHg |  |  |  |  |  |
| - At beginning of follow-up | 68 +/- 4 | 77 +/- 4 | 86 +/- 4 | 96 +/- 6 | <.001 |
| - At end of follow-up | 70 +/- 4 | 76 +/- 5 | 81 +/- 5 | 88 +/- 7 | <.001 |
| DBP current*, mean +/- SD, mmHg |  |  |  |  |  |
| - At beginning of follow-up | 66 +/- 6 | 76 +/- 6 | 86 +/- 7 | 100 +/- 11 | <.001 |
| - At end of follow-up | 70 +/- 8 | 75 +/- 9 | 77 +/- 10 | 79 +/- 14 | <.001 |
| LDL age 20-39, mean +/- SD, mg/dl | 107 +/- 20 | 119 +/- 23 | 131 +/- 22 | 141 +/- 22 | <.001 |
| LDL age 40+, mean +/- SD, mg/dl |  |  |  |  |  |
| - At beginning of follow-up | 115 +/- 25 | 127 +/- 28 | 138 +/- 27 | 144 +/- 28 | <.001 |
| - At end of follow-up | 121 +/- 25 | 129 +/- 25 | 132 +/- 24 | 134 +/- 26 | <.001 |
| LDL current*, mean +/- SD, mg/dl |  |  |  |  |  |
| - At beginning of follow-up | 112 +/- 28 | 125 +/- 33 | 137 +/- 34 | 144 +/- 35 | <.001 |
| - At end of follow-up | 123 +/- 32 | 126 +/- 32 | 125 +/- 33 | 121 +/- 35 | <.001 |
| HDL age 20-39, mean +/- SD, mg/dl | 55 +/- 9 | 50 +/- 10 | 44 +/- 9 | 41 +/- 7 | <.001 |
| HDL age 40+, mean +/- SD, mg/dl |  |  |  |  |  |
| - At beginning of follow-up | 57 +/- 11 | 52 +/- 12 | 46 +/- 11 | 42 +/- 9 | <.001 |
| - At end of follow-up | 58 +/- 13 | 52 +/- 13 | 46 +/- 12 | 42 +/- 9 | <.001 |
| HDL current*, mean +/- SD, mg/dl |  |  |  |  |  |
| - At beginning of follow-up | 56 +/- 13 | 52 +/- 14 | 47 +/- 14 | 44 +/- 12 | <.001 |
| - At end of follow-up | 60 +/- 16 | 54 +/- 16 | 47 +/- 15 | 43 +/- 14 | <.001 |

* - Current values are the most recent direct measurements, with the last value carried forward.

SBP – Systolic blood pressure; DBP – Diastolic blood pressure; LDL – Low-density lipoprotein cholesterol; HDL – High-density lipoprotein cholesterol

| **Appendix Table D. Characteristics of participants at the beginning of follow up, stratified by early life exposure to HDL** | | | | | |
| --- | --- | --- | --- | --- | --- |
| Characteristics | Time-weighted average HDL from age 20-39, mg/dl | | | | p-value |
|  | >65  N=328 | 51-65  N=1673 | 36-50  N=2494 | <=35  N=365 |  |
| Sex, n (%) male | 19 (6%) | 343 (21%) | 1637 (66%) | 332 (91%) | <.001 |
| Year, mean +/- SD |  |  |  |  |  |
| - At beginning of follow-up | 1982 +/- 8 | 1980 +/- 7 | 1979 +/- 7 | 1979 +/- 7 | <.001 |
| - At end of follow-up | 2005 +/- 5 | 2004 +/- 6 | 2002 +/- 8 | 2000 +/- 10 | <.001 |
| Age, mean +/- SD, years |  |  |  |  |  |
| - At beginning of follow-up | 41 +/- 3 | 42 +/- 4 | 43 +/- 5 | 43 +/- 5 | <.001 |
| - At end of follow-up | 65 +/- 10 | 66 +/- 10 | 66 +/- 10 | 64 +/- 10 | <.001 |
| Body mass index, mean +/- SD, kg/m^2^ |  |  |  |  |  |
| - At beginning of follow-up | 24 +/- 4 | 25 +/- 4 | 28 +/- 4 | 29 +/- 4 | <.001 |
| - At end of follow-up | 28 +/- 5 | 29 +/- 6 | 30 +/- 5 | 31 +/- 5 | <.001 |
| Diabetes, n (%) |  |  |  |  |  |
| - At beginning of follow-up | 1 (0.3%) | 16 (1%) | 74 (3%) | 22 (6%) | <.001 |
| - At end of follow-up | 16 (5%) | 101 (6%) | 340 (14%) | 98 (27%) | <.001 |
| Diabetes duration, mean +/- SD, years |  |  |  |  |  |
| - At beginning of follow-up | .02 +/- .44 | .06 +/- .64 | .17 +/- 1.1 | .29 +/- 1.6 | <.001 |
| - At end of follow-up | .66 +/- 3.3 | .96 +/- 4.3 | 2.1 +/- 6 | 3.8 +/- 8 | <.001 |
| Current smoking, n (%) |  |  |  |  |  |
| - At beginning of follow-up | 128 (39%) | 713 (43%) | 1395 (56%) | 243 (67%) | <.001 |
| - At end of follow-up | 22 (7%) | 151 (9%) | 330 (13%) | 90 (25%) | <.001 |
| Pack-years tobacco exposure, mean +/- SD |  |  |  |  |  |
| - At beginning of follow-up | 9 +/- 14 | 11 +/- 15 | 18 +/- 20 | 23 +/- 21 | <.001 |
| - At end of follow-up | 12 +/- 19 | 15 +/- 21 | 24 +/- 27 | 31 +/- 28 | <.001 |
| Blood pressure medication, n (%) |  |  |  |  |  |
| - At beginning of follow-up | 16 (5%) | 102 (6%) | 183 (7%) | 41 (11%) | .002 |
| - At end of follow-up | 80 (24%) | 485 (29%) | 967 (39%) | 187 (51%) | <.001 |
| Lipid medication use, n (%) |  |  |  |  |  |
| - At beginning of follow-up | 1 (0.3%) | 6 (0.4%) | 17 (0.7%) | 10 (3%) | <.001 |
| - At end of follow-up | 13 (4%) | 167 (10%) | 420 (17%) | 99 (27%) | <.001 |
| SBP age 20-39, mean +/- SD, mmHg | 114 +/- 9 | 116 +/- 9 | 123 +/- 10 | 127 +/- 9 | <.001 |
| SBP age 40+, mean +/- SD, mmHg |  |  |  |  |  |
| - At beginning of follow-up | 116 +/- 12 | 118 +/- 12 | 124 +/- 12 | 127 +/- 11 | <.001 |
| - At end of follow-up | 123 +/- 13 | 126 +/- 13 | 129 +/- 12 | 131 +/- 11 | <.001 |
| SBP current*, mean +/- SD, mmHg |  |  |  |  |  |
| - At beginning of follow-up | 116 +/- 16 | 118 +/- 15 | 124 +/- 16 | 127 +/- 15 | <.001 |
| - At end of follow-up | 125 +/- 20 | 128 +/- 19 | 130 +/- 18 | 131 +/- 18 | <.001 |
| DBP age 20-39, mean +/- SD, mmHg | 73 +/- 6 | 75 +/- 6 | 80 +/- 7 | 83 +/- 6 | <.001 |
| DBP age 40+, mean +/- SD, mmHg |  |  |  |  |  |
| - At beginning of follow-up | 75 +/- 8 | 77 +/- 7 | 81 +/- 8 | 84 +/- 7 | <.001 |
| - At end of follow-up | 74 +/- 6 | 76 +/- 6 | 79 +/- 6 | 81 +/- 6 | <.001 |
| DBP current*, mean +/- SD, mmHg |  |  |  |  |  |
| - At beginning of follow-up | 75 +/- 10 | 77 +/- 10 | 81 +/- 11 | 84 +/- 11 | <.001 |
| - At end of follow-up | 73 +/- 10 | 74 +/- 9 | 76 +/- 10 | 78 +/- 11 | <.001 |
| LDL age 20-39, mean +/- SD, mg/dl | 105 +/- 20 | 114 +/- 22 | 128 +/- 23 | 132 +/- 25 | <.001 |
| LDL age 40+, mean +/- SD, mg/dl |  |  |  |  |  |
| - At beginning of follow-up | 112 +/- 24 | 123 +/- 26 | 137 +/- 27 | 138 +/- 30 | <.001 |
| - At end of follow-up | 118 +/- 25 | 126 +/- 25 | 133 +/- 24 | 129 +/- 28 | <.001 |
| LDL current*, mean +/- SD, mg/dl |  |  |  |  |  |
| - At beginning of follow-up | 108 +/- 28 | 119 +/- 32 | 136 +/- 33 | 136 +/- 36 | <.001 |
| - At end of follow-up | 116 +/- 33 | 124 +/- 32 | 127 +/- 32 | 120 +/- 36 | <.001 |
| HDL age 20-39, mean +/- SD, mg/dl | 70 +/- 4 | 56 +/- 4 | 43 +/- 4 | 32 +/- 2 | <.001 |
| HDL age 40+, mean +/- SD, mg/dl |  |  |  |  |  |
| - At beginning of follow-up | 74 +/- 7 | 59 +/- 6 | 43 +/- 5 | 31 +/- 3 | <.001 |
| - At end of follow-up | 75 +/- 10 | 59 +/- 9 | 44 +/- 7 | 32 +/- 5 | <.001 |
| HDL current*, mean +/- SD, mg/dl |  |  |  |  |  |
| - At beginning of follow-up | 79 +/- 13 | 60 +/- 9 | 44 +/- 7 | 30 +/- 5 | <.001 |
| - At end of follow-up | 77 +/- 16 | 61 +/- 13 | 45 +/- 10 | 33 +/- 8 | <.001 |

* - Current values are the most recent direct measurements, with the last value carried forward.

SBP – Systolic blood pressure; DBP – Diastolic blood pressure; LDL – Low-density lipoprotein cholesterol; HDL – High-density lipoprotein cholesterol

| **Appendix Table E. Coronary Heart Disease Events in Framingham Participants with Differing Exposure to Risk Factors During Young Adulthood, limited to participants with at least one direct risk factor measurement before age 40** | | | | | | | |
| --- | --- | --- | --- | --- | --- | --- | --- |
| Time-weighted average from age 20-39 | N | Coronary Heart Disease Events | | | | | |
|  |  | N (%) with an event | Total person-time observed | Unadjusted event rate, per 1000 person-years | Hazard ratio (95% confidence interval) | | |
|  |  |  |  |  | Unadjusted | Adjusted for other risk factors* | Additionally adjusted for later life exposure*† |
| SBP, mmHg |  |  |  |  |  |  |  |
| <=120 | 1,855 | 38 (2.0%) | 41,356 | 0.9 | 1 (reference) | 1 (reference) | 1 (reference) |
| 121-140 | 1,081 | 126 (12%) | 25,110 | 5.0 | 5.3 (3.7-7.7) | 1.8 (1.1-3.0) | 1.3 (0.7-2.3) |
| 141-160 | 33 | 5 (15%) | 735 | 6.8 | 7.3 (2.9-18) | 0.8 (0.2-2.7) | 0.8 (0.2-2.6) |
| >160 | 0 | 0 | 0 | --- | --- | --- | --- |
|  |  |  |  | Trend p-value | <.001 | .74 | .67 |
|  |  |  |  |  |  |  |  |
| DBP, mmHg |  |  |  |  |  |  |  |
| <=70 | 589 | 5 (0.8%) | 12,245 | 0.4 | 1 (reference) | 1 (reference) | 1 (reference) |
| 71-80 | 1,649 | 61 (3.7%) | 38,078 | 1.6 | 3.7 (1.5-9.3) | 1.6 (0.6-4.2) | 1.5 (0.5-4.8) |
| 81-90 | 674 | 91 (14%) | 15,632 | 5.8 | 13 (5.5-33) | 2.9 (1.0-8.0) | 2.6 (0.8-8.8) |
| >90 | 57 | 12 (21%) | 1,246 | 9.6 | 23 (8.0-65) | 5.9 (1.6-22) | 4.4 (1.0-19) |
|  |  |  |  | Trend p-value | <.001 | .004 | .032 |

| LDL, mg/dl |  |  |  |  |  |  |  |
| --- | --- | --- | --- | --- | --- | --- | --- |
| <=100 | 731 | 7 (1.0%) | 16,123 | 0.4 | 1 (reference) | 1 (reference) | 1 (reference) |
| 101-130 | 1,453 | 62 (4.3%) | 33,236 | 1.9 | 4.2 (1.9-9.2) | 2.5 (1.1-5.6) | 2.1 (0.8-5.4) |
| 131-160 | 663 | 79 (12%) | 15,032 | 5.2 | 12 (5.5-26) | 4.8 (2.2-10) | 3.4 (1.2-9.6) |
| >160 | 123 | 21 (17%) | 2,810 | 7.4 | 17 (7.1-40) | 4.6 (1.9-11) | 3.0 (0.9-10) |
|  |  |  |  | Trend p-value | <.001 | <.001 | .054 |
|  |  |  |  |  |  |  |  |
| HDL, mg/dl |  |  |  |  |  |  |  |
| >65 | 247 | 2 (0.8%) | 5,517 | 0.4 | 1 (reference) | 1 (reference) | 1 (reference) |
| 51-65 | 1,084 | 24 (2.2%) | 24,849 | 1.0 | 2.6 (0.6-11) | 1.2 (0.3-5.3) | 0.9 (0.2-4.4) |
| 36-50 | 1,422 | 112 (7.9%) | 32,118 | 3.5 | 9.5 (2.3-39) | 2.0 (0.5-8.3) | 0.8 (0.2-4.2) |
| <=35 | 216 | 31 (14.4%) | 4,717 | 6.6 | 18 (4.3-76) | 2.1 (0.5-9.5) | 0.9 (0.4-4.9) |
|  |  |  |  | Trend p-value | <.001 | .25 | .84 |

* - All multivariable models adjusted for age (via Cox model), sex, calendar year (via spline), body mass index, diabetes, years with diabetes, smoking status (current/past/never), pack-years of tobacco exposure (via spline), and use of blood pressure and lipid medications. Models are also adjusted for the *other* risk factors in the table; for example, each adjusted SBP model is adjusted for all the DBP, LDL, HDL predictors including both early and later life exposure.

† - Adjusted for measurements of each risk factor made later in life, including the time-weighted average from age 40+ as well as the most recent measurement (with the last value carried forward), categorized in the same way. For example, the LDL hazard ratios in the last column are adjusted for all other risk factors* as well as LDL exposure from age 40+ as well as the most recent LDL level.

SBP – Systolic blood pressure; LDL – Low-density lipoprotein cholesterol; HDL – High-density lipoprotein cholesterol

| **Appendix Table F. Sensitivity analysis results using continuous risk factors** | | | | |
| --- | --- | --- | --- | --- |
| Time-weighted average from age 20-39 | Hazard ratio (95% confidence interval) for coronary heart disease events, adjusted for all other risk factors* | | | |
|  | A  In full sample, adjusted for all covariates as in Table 3 right column  N=4860 | B  In limited sample including only participants with at least one direct risk factor measurement before age 40  N=2969 | C  B, plus dropping the time-weighted average measurements after age 40 but keeping the last value carried forward*  N=2969 | D  C, dropping early life exposure to systolic blood pressure*  N=2969 |
| SBP, per 20 mmHg | 1.1 (0.6-2.0) | 2.1 (0.5-8.0) | 0.7 (0.2-1.7) | --- |
| DBP, per 10 mmHg | 1.6 (1.1-2.3) | 1.8 (0.8-3.8) | 2.2 (1.2-4.1) | 1.8 (1.2-2.6) |
| LDL, per 30 mg/dl | 1.5 (1.1-2.0) | 1.3 (0.8-2.2) | 1.5 (1.1-2.0) | 1.5 (1.1-2.0) |
| HDL, per 15 mg/dl | 1.2 (0.7-1.9) | 1.6 (0.7-3.3) | 1.0 (0.6-1.5) | 0.9 (0.6-1.5) |

* - This strategy was undertaken due to evidence of collinearity in the continuous predictor models. A collinearity analysis for the variables included in Sensitivity Analysis B, for example, showed variance inflation factors (VIFs) over 10 for all later-life time-weighted average risk factor measurements. Removing these variables from the model, while still including the last later life risk factor measurement carried forward (Sensitivity Analysis C) produced VIF’s all below 10, though the early life SBP and DBP variables were still high (9.1 for SBP and 7.2 for DBP). Additionally removing early life SBP from the model (Sensitivity Analysis D) yielded similar results without residual evidence of collinearity (VIFs all less than 4). Removing early life DBP from the model instead of early life SBP (not shown in this table) yielded a non-significant hazard ratio of 1.6 (0.8-2.9) for early adult SBP, consistent with our other results.

Appendix Figure Legend

**Appendix Figure. Individual trajectories of body mass index, systolic and diastolic blood pressure, lipids, and diabetes onset for selected Framingham participants**

Body mass index (BMI, in kg/m2), systolic and diastolic blood pressure (SBP and DBP, in mg/dl), high density and low density lipoprotein cholesterol (HDL and LDL cholesterol, in mg/dl), and diabetes (DM, presence/absence) are plotted by age, starting at age 20 and ending at the end of their follow up time in Framingham. Trajectories were fit using random effects models with age modeled as a cubic spline, including random intercepts and spline components by participant, as well as a random effect offset for presence of diabetes and medication use for lipids and blood pressure. These methods allow for imputation of the trajectory before and between measured values of each risk factor. See Methods for details. We have selected 5 participants who never developed diabetes or used any lipid or blood pressure medications (Panel 1), 5 participants who used lipid medications (Panel 2), 5 participants who used blood pressure medications (Panel 3), and 5 participants who developed diabetes (Panel 4) to illustrate the range of trajectories, degree of imputation and extrapolation back to age 20, and range of effects from diabetes and medication use fit from our modeling strategy.

**Appendix Figure, Panel 1:** Participants who never developed diabetes or used any lipid or blood pressure medications

**
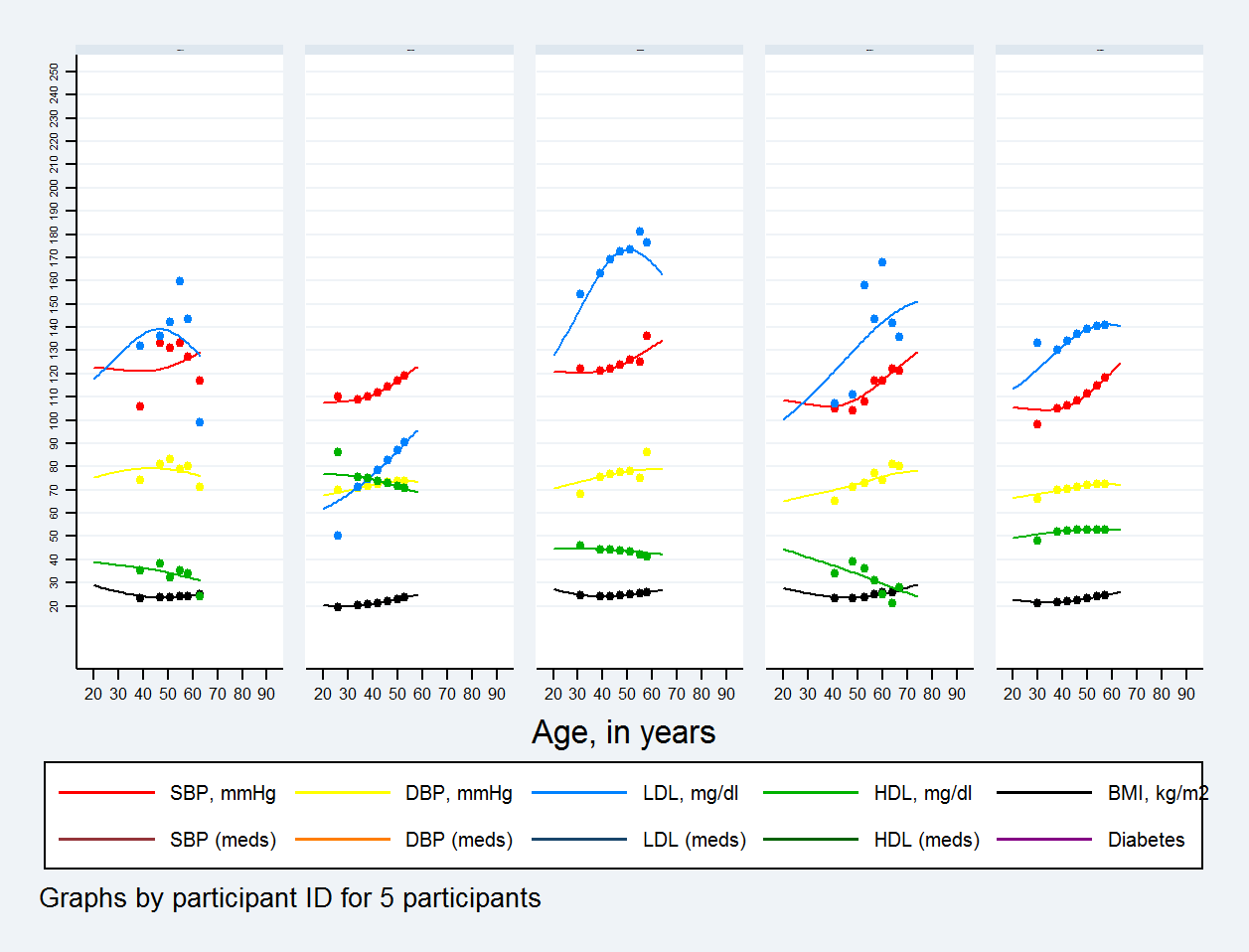
**

**Appendix Figure, Panel 2:** Participants who used lipid medications


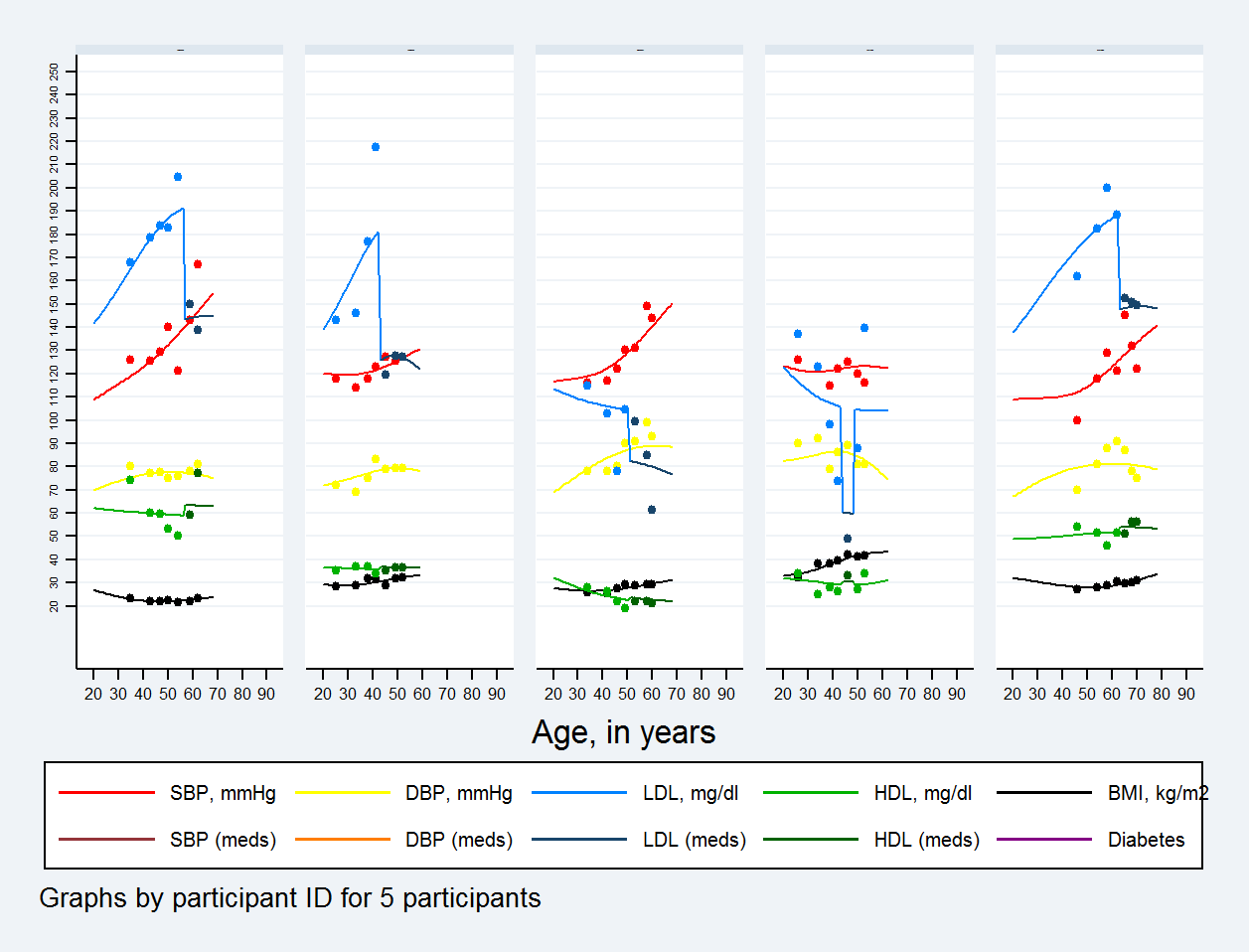


**Appendix Figure, Panel 3:** Participants who used blood pressure medications


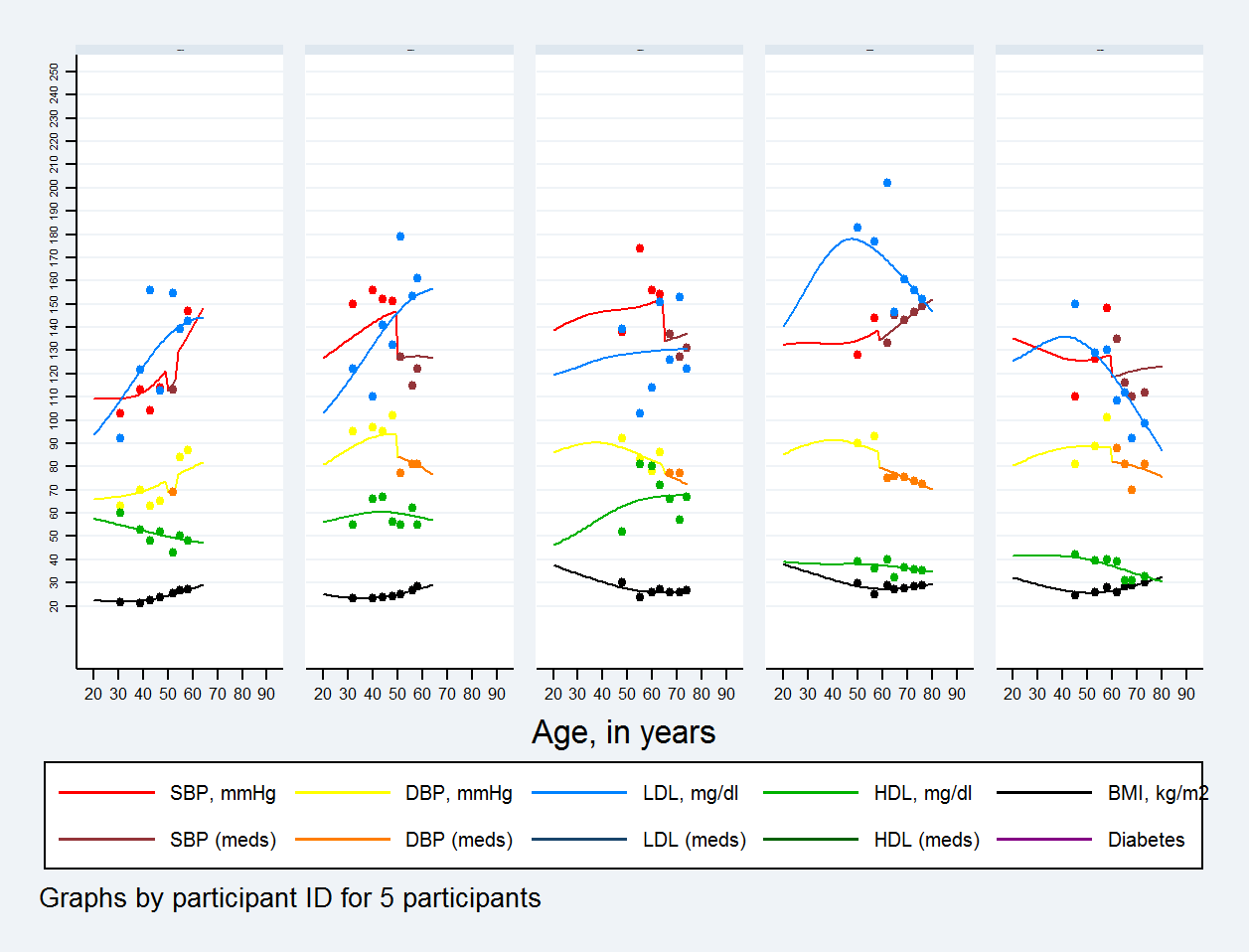


**Appendix Figure, Panel 4:** Participants who developed diabetes


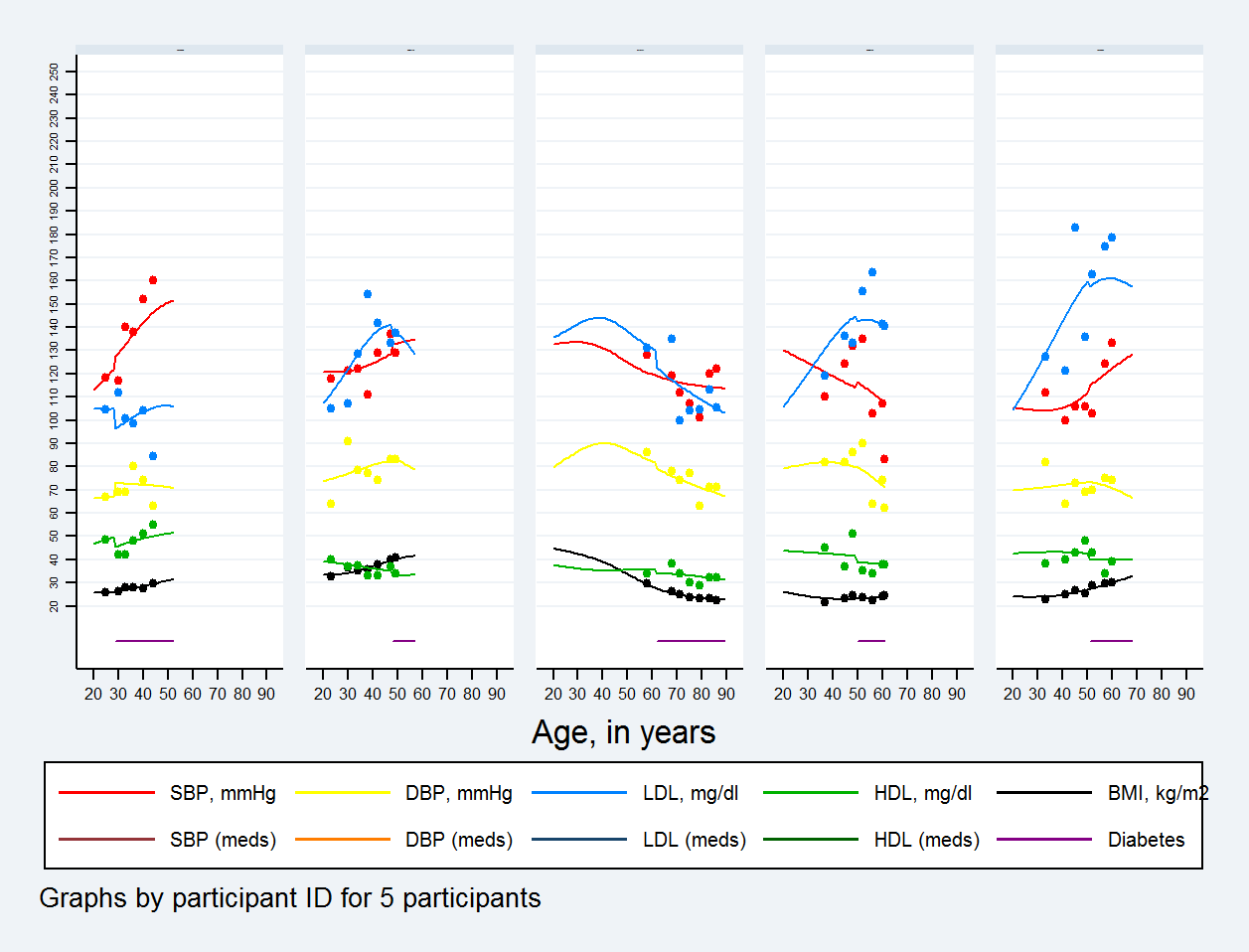

Supplement: S1 Appendix — (DOC) [file pone.0154288.s001.doc]
